# Does the Relation Between Humor Styles and Subjective Well-Being Vary Across Culture and Age? A Meta-Analysis

**DOI:** 10.3389/fpsyg.2020.02213

**Published:** 2020-09-22

**Authors:** Feng Jiang, Su Lu, Tonglin Jiang, Heqi Jia

**Affiliations:** ^1^Department of Organization and Human Resources Management, Central University of Finance and Economics, Beijing, China; ^2^Division of Psychology, De Montfort University, Leicester, United Kingdom; ^3^School of Psychological and Cognitive Sciences and Beijing Key Laboratory of Behavior and Mental Health, Peking University, Beijing, China; ^4^Department of Human Resource and Organizational Behavior, University of International Business and Economics, Beijing, China

**Keywords:** age, culture, humor styles, subjective well-being, meta—analysis

## Abstract

An earlier review (Schneider et al., 2018) examined the connection between humor styles and mental health. The present article supplements and extends Schneider et al.'s review by surveying a broader concept, subjective well-being (SWB), and investigating the moderating effects of culture and age. To this end, we collected data from 85 studies, with 27,562 participants of varying ages and cultures. Meta-analysis results indicate that affiliative and self-enhancing humor enhances SWB, whereas aggressive and self-defeating humor damages SWB. Culture and age do not moderate the relation between humor styles and SWB. We discuss implications for better understanding of the relationships among culture, age, humor, and SWB.

The past decades have witnessed a burgeoning interest in the study of the psychology of humor (Martin and Ford, 2018). Psychologists tend to view humor as a positive, desirable enhancement to subjective well-being (SWB), which is commonly assessed along the lines of satisfaction, happiness, or quality of life. For example, past research suggests that humor relieves stress, tension, anxiety, and depression (e.g., Abe, 1994; Kuiper and Martin, 1998; Lefcourt, 2001; Nezlek and Derks, 2001; Martin and Ford, 2018). Studies also show humor is associated with optimism, autonomy, positive self-concepts, good social relationships, positive affect, and life satisfaction (e.g., Kuiper and Martin, 1993, 1998; Kuiper and Olinger, 1998; Parrish and Quinn, 1999; Lefcourt, 2001; Nezlek and Derks, 2001; Abel, 2002; Yip and Martin, 2006; Martin and Ford, 2018).

However, empirical findings in some studies do not support the positive relation between humor and SWB. For example, humor was not found to be significantly associated with self-acceptance, positive interpersonal relationships, autonomy, environmental mastery, and purpose in life (Svebak, 1974; Lefcourt and Martin, 1986; Ryff, 1989; Nezlek and Derks, 2001).

One reason explaining these mixed findings might be that previous research failed to differentiate between potentially beneficial and detrimental styles of humor. Humor styles represent the ways individuals use humor as a strategy for coping as well as shifting their perspectives (Dozois et al., 2009). In other words, different humor styles may play constructive or destructive roles in one's mental health. Martin et al. (2003) differentiated four humor styles: (a) *self-enhancing humor* is used to augment oneself; (b) *affiliative humor* is used to maintain and enhance interpersonal relationships; (c) *aggressive humor* is used to enhance oneself at the expense of others; (d) *self-defeating humor* is used for self-deprecation or self-disparagement. Among the four humor styles, self-enhancing, and affiliative humor styles are commonly regarded as adaptive humor styles, whereas aggressive and self-defeating humor styles are treated as maladaptive humor styles (Dozois et al., 2009). More importantly, these humor styles are found in Eastern and Western cultures, suggesting a cross-cultural universality (Saroglou and Scariot, 2002; Martin et al., 2003; Chen and Martin, 2007; Taher et al., 2008).

Generally speaking, adaptive humor styles (i.e., affiliative and self-enhancing) are positively associated with SWB, whereas maladaptive humor styles (i.e., aggressive and self-defeating) are negatively associated with SWB (e.g., Martin et al., 2003; Kuiper et al., 2004). To be specific, affiliative humor promotes adjustment, relieves anxiety, and counters depression (e.g., Chen and Martin, 2007; Frewen et al., 2008), whereas self-enhancing humor promotes optimism and self-esteem and deactivates depression (Chen and Martin, 2007; Dozois et al., 2009; Martin and Ford, 2018). In contrast, aggressive humor is associated with lower self-esteem, higher loneliness, and aggression (e.g., Martin et al., 2003; Kuiper et al., 2004; Kazarian and Martin, 2006; Cann et al., 2008), whereas self-defeating humor is associated with higher depression and anxiety (e.g., Martin et al., 2003; Chen and Martin, 2007; Cann et al., 2008; Martin and Ford, 2018).

The above associations were mainly verified in Western cultures; nonetheless, despite the universality of the four humor styles, people from different cultures react differently to each. For example, because Chinese culture stresses harmony and peace, Chinese students tend to use aggressive humor less often as a coping strategy in comparison with Canadian students (Chen and Martin, 2007). Hong Kong has experienced weaker collectivism influences than mainland China. A comparison of students from mainland China and Hong Kong showed that Hong Kong students tend toward aggressive and self-defeating humor and away from affiliative and self-enhancing humor (Yue et al., 2014b, 2016b). A study of cross-countries samples found that individuals from horizontal collectivist cultures are more likely to use affiliative humor to foster interdependence; individuals from vertical collectivist cultures are more likely to use self-defeating humor for the sake of the group; and individuals from vertical individualist cultures are more likely to use aggressive humor to enhance their hierarchical status (Kazarian and Martin, 2004). In short, it seems that people from Western culture are apt to use self-defeating and aggressive humor, whereas people from Eastern culture tend to embrace self-enhancing and affiliative humor (e.g., Abe, 1994; Nevo et al., 2001; Chen and Martin, 2005, 2007; Liao and Chang, 2006; Yue, 2011).

Thus, the imperative question arises of whether the humor–SWB relationship found primarily in Western culture still holds in Eastern culture. To address this issue, we first review cultural differences in humor perception and usage.

## Humor and SWB in Different Cultures

People from Eastern collectivistic cultures value harmony and peace, treasure doctrine and hierarchy, and emphasize interdependence over independence. Therefore, they diverge from Westerners such that they consider humor to be an undesirable trait and a poor coping strategy (e.g., Chen and Martin, 2007). However, empirical studies concerning humor effects are mixed. On one hand, numerous studies suggest a great similarity between humor effects between the East and the West; that is, like Westerners, Easterners perceive that self-enhancing and affiliative humor contribute to SWB and that aggressive and self-defeating humor damages SWB. For example, Chinese students indicated humor that is affiliative and self-enhancing is positively associated with increased self-esteem, self-compassion, and optimism, as well as with decreased loneliness and distress (Sun et al., 2009; Cheung and Yue, 2013; Yue et al., 2014b, 2017). Similarly, the use of aggressive and self-defeating humor was positively correlated with loneliness, depression, anxiety, and lowered self-esteem (Sun et al., 2009; Cheung and Yue, 2013; Yue et al., 2014a,b). Similarly, a study comparing Chinese and Canadian students found no cultural differences in the relationship between humor and SWB (Chen and Martin, 2007).

On the other hand, recent studies show that Easterners may perceive and use humor differently from Westerners. Specifically, Easterners' attitudes toward humor are not as positive as Westerners' are. For examples, Mainland Chinese students showed no significant difference from American students in explicit attitudes toward humor; however, the former associated humor more frequently with unpleasant adjectives and seriousness with pleasant adjectives on the implicit attitude, but the opposite pattern was found for their American counterparts (Jiang et al., 2011). Similarly, they do not regard humor as an indicator of creativity in the way Westerners do (Rudowicz and Yue, 2002; Yue and Hui, 2015; Kellner and Benedek, 2017). More compellingly, they perceive humor as less important and rate themselves as being less humorous than Western counterparts are (Chen and Martin, 2005). Yue et al. (2016a) asked participants to nominate up to three very funny persons and to identify the occupations of the humorists. Results indicated that compared to Chinese participants, Canadian participants nominated more relatives and friends and their nominees had much broader occupations. Yue et al. (2016a) work suggests that Westerners may expect ordinary people to possess humor, whereas Chinese people usually do not expect comedy from non-professional comedians. Little research has been conducted or published in English or Chinese with respect to the humor effect in Africa, Latin America, and Middle East.

Given the reported considerable differences concerning cultural attitudes toward humor and its association with SWB, it is important to gain better insight into the potential influence of culture.

## Age Effects on Humor and SWB Relation

Another issue that lacks consensus in humor psychology is the role of age. There is no consensus on how age influences the humor–SWB relationship. The first problem is that some researchers emphasize age differences. For example, studies show younger individuals are more likely to use aggressive, self-defeating humor as a coping strategy than older individuals are (Martin et al., 2003; Kazarian and Martin, 2006; Chen and Martin, 2007). However, other research suggests there are no age differences in humor usage (e.g., Tümkaya, 2011; Liu, 2012). In studies involving children and adolescents as samples, researchers have used the adult-standardized Humor Styles Questionnaire to show that humor affects children and adolescents in a way that is similar to adults; that is, adaptive rather than maladaptive humor is positively associated with SWB (McGhee, 1980; McGhee and Chapman, 1980; Bell et al., 1986; Carson et al., 1986; Erickson and Feldstein, 2007).

Research on age differences in general coping mechanisms may shed light on this inconsistency. McCrae (1989) summarized three competing hypotheses relating to age differences in coping strategies. The *regression hypothesis* argues that individuals would start to use more defensive (vs. adaptive) coping strategies as they come into the later stage of their lives (Gutmann, 1974). Hence, a decreasing use of adaptive humor styles and an increasing use of maladaptive humor styles is expected as individuals grow older. However, the *growth hypothesis* suggests that as individuals grow older, they are more apt to employ adaptive coping strategies (Vaillant, 1977). The *contextual hypothesis* asserts that the influence of age on coping strategies depends upon contextual factors, such as different problems people face at different developmental stages (e.g., Folkman and Lazarus, 1980; McCrae, 1984). Therefore, the contextual hypothesis suggests no systematic relationship between age and humor styles usage.

In summary, both theoretical assumptions and empirical findings have shown mixed outcomes. It is worth investigating how age influences the relationships between humor styles and SWB. As a compelling and popular topic in positive psychology, the mechanisms by which humor affects SWB attract much attention (Kuiper et al., 1992; Kuiper and Martin, 1998; Martin, 2001, 2002; Celso et al., 2003; Zhao et al., 2012). Although humor is universal, it may have varying effects on SWB in different cultures and for individuals at different ages (Fry, 1994; Martin and Ford, 2018). Thus, we undertook this research primarily to investigate how humor styles and the roles of culture and age are associated with SWB.

## The Current Research

Schneider et al. (2018) conducted a meta-analysis of 37 studies involving samples of 12,734 participants. The covered publication period is from 2003 to 2015. In the research, they investigated the association between the four humor styles and mental health, indexed by self-esteem, life satisfaction, optimism, and depression. They found that affiliative and self-enhancing humor styles positively associated with the indices of mental health and self-defeating humor styles negatively associated with the indices of mental health. No significant association was found between aggressive humor style and mental health index. They also found that culture and gender moderate the effects of humor. An aggressive humor style negatively associated with self-esteem and positively associated with depression for Easterners, but not for Westerners. The association between self-enhancing humor style and optimism is quite lower in Asian samples in comparison with North American and European samples. Women were found have a larger affiliative humor style and optimism association, and a smaller affiliative humor style and life satisfaction association in comparison with men (Schneider et al., 2018). Although Schneider et al. provided evidence of interconnections between humor styles and mental health, it is still necessary to conduct a new meta-analysis for two reasons. First, their work investigated a relative narrow scope of mental health (i.e., self-esteem, life satisfaction, optimism, and depression). However, SWD is a broader concept that includes “both cognitive judgments of one's life satisfaction in addition to affective evaluations of mood and emotions” (Diener and Lucas, 1999, p. 213). For the most part, categories of SWB can be roughly identified as self-esteem, life satisfaction, optimism, affect, and happiness (e.g., Lucas et al., 1996; DeNeve and Cooper, 1998). However, the indices used to indicate these categories appeared to be inclusive. For example, life satisfaction was indexed with job satisfaction (Weiss, 2002), and affect was represented by anxiety (Spielberger et al., 1970), extraversion, neuroticism, and optimism (Steel et al., 2008). Echoing the notion that SWB is a multifaceted concept, in the current meta-analysis, we investigated the effects of humor styles on SWB with a wider scope. Second, the focus on mental health narrowed down Schneider et al. (2018) range of studies available for selection; thus, the moderating effects (culture in particular) were tested with insufficient sample sizes—the value of *k* (number of studies) was too small, which may result in significant bias in interpreting the outcomes (Field and Gillett, 2010).

To sum up, this meta-analysis seeks to address two major questions: (a) Do significant correlations exist between humor styles and SWB, and if so, what is the size of these relations? (b) Do culture and age affect the relations between humor styles and SWB? The present study aims to replicate previous findings with an updated corpus of studies and address the questions stated above. To identify relevant studies examining humor styles and SWB, we searched several databases covering research published from 2003 to 2019. We isolated 85 studies that strictly concern the relationship between humor styles and SWB. Following Hunter and Schmidt (1990), we conducted a meta-analysis to test the relationship between humor and SWB and the moderating roles of culture and age.

## Method

### Rules for Inclusion in the Meta-Analysis

To identify relevant studies examining humor styles and SWB, we searched several databases covering studies published from 2003 to 2019, including PsycINFO, Google Scholar, and ProQuest (unpublished dissertations). We chose 2003 as our start point because it was then that Martin et al. (2003) developed the Humor Styles Questionnaire.

Recall that self-enhancing and affiliative humor styles are commonly regarded as positive or adaptive, whereas aggressive and self-defeating humor styles are regarded as negative or maladaptive (e.g., Dozois et al., 2009). As an extension, we incorporated positive, negative, adaptive, and maladaptive humor styles into Martin et al.'s four humor styles. In conjunction with our focus on SWB, we used seven key search words: *humor, humor styles questionnaire, humor categories, positive humor, negative humor, adaptive humor*, and *maladaptive humor*.

**Figure 1:**
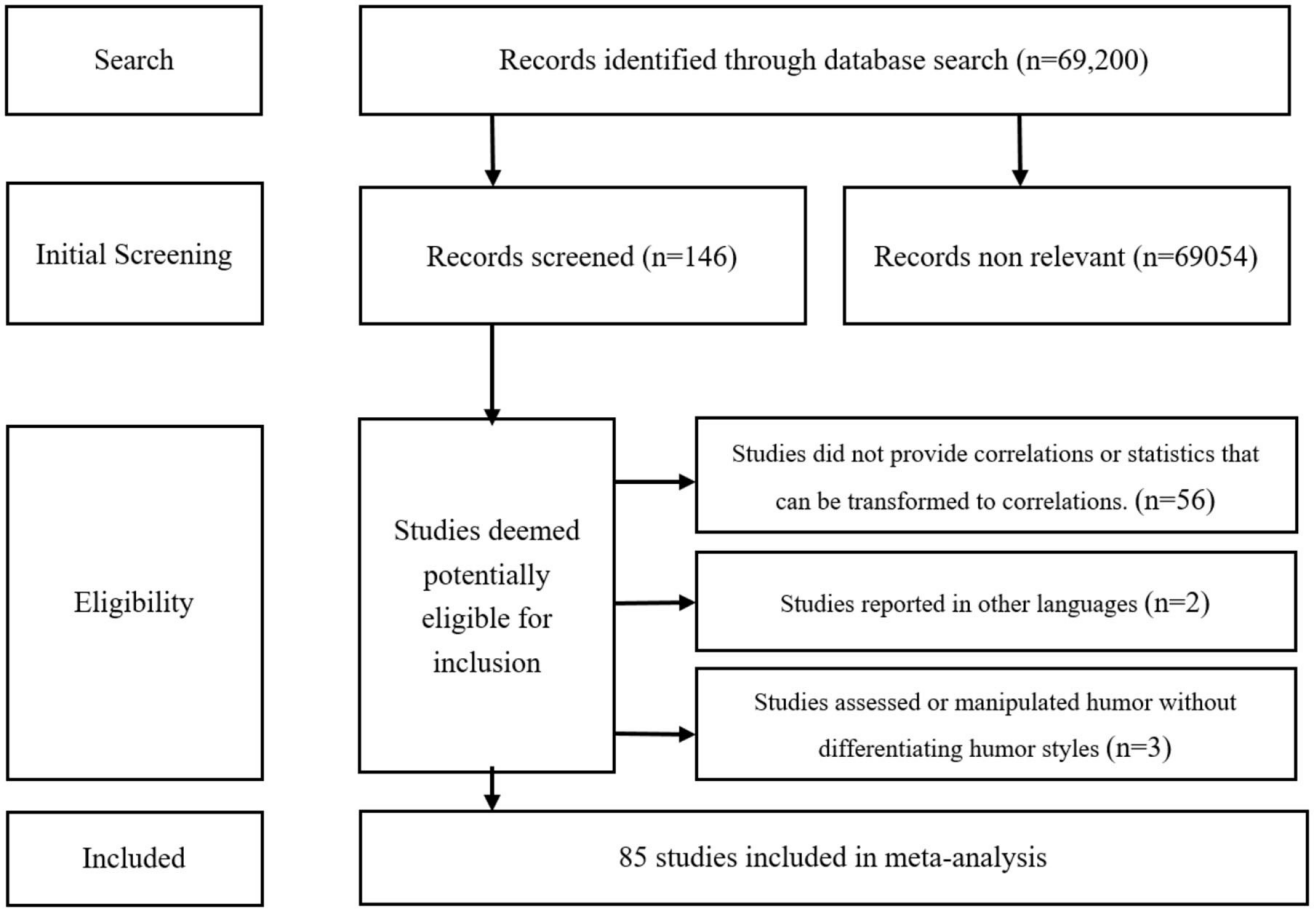
Study search and inclusion criteria flow diagram.

The initial search yielded 69,200 studies. We then reviewed all studies by titles and abstracts, excluding 69,054 irrelevant papers. Thus, we identified 146 published articles, dissertations, and book chapters. We then conducted a full paper sift of each study using the specific criteria described below. Figure 1 displays the flow diagram for the search and inclusion criteria.

First, studies had to report at least one correlation between humor styles and any measure of SWB, or any statistics that can be transformed to correlations. The measures of humor styles and SWB could be administered at the same time or different times. Second, studies had to include assessment of the four humor styles, or any category such as adaptive and maladaptive humor. Those studies investigating the relationship between humor and SWB were still excluded if they did not differentiate humor styles. For example, we excluded some studies that measured sense of humor, humor production, humor tolerance, and importance of humor (e.g., Yue et al., 2016a). Third, study designs could be cross-sectional, experimental, or randomized (or non-randomized) controlled trials. Publications such as case reports, editorial and opinion pieces, and book reviews were not included. In addition, experimental studies without differentiating humor in terms of different types were also excluded. Fourth, due to the limits of our research team, we only included studies written in English or Chinese. Of all the studies selected, Chinese articles comprised only 3.5%.

We obtained 85 valid studies strictly focusing on the relation between humor styles and SWB with different indices. Consistent with our focus, the studies identified affiliative, self-enhancing, aggressive, self-defeating, positive, negative, adaptive, and maladaptive styles of humor. SWB measures included anxiety, distress, subjective happiness, stress, positive affect, negative affect, depression, optimism, self-esteem, life satisfaction, school satisfaction, job satisfaction, loneliness, extraversion, neuroticism, flourishing, and so forth. The measurements are highly consistent with categories identified previously (e.g., Lucas et al., 1996; DeNeve and Cooper, 1998; Connolly and Viswesvaran, 2000; Thoresen et al., 2003). As a result, we recorded 1,216 effect sizes regarding the relationship between humor styles and SWB. Many shared the same sample size in a single study, so we followed Hunter and Schmidt (2004) suggestion to aggregate them to avoid inflating the sample size. Two research assistants independently coded humor styles as adaptive or maladaptive and coded SWB as positive or negative. The interrater reliability was 1 for humor styles and 1 for SWB. Thus, within each study, we created 2 humor styles (adaptive vs. maladaptive) × 2 SWB (positive vs. negative) relationships with one effect size for each relationship. These four associations were analyzed separately.

### Meta-Analytic Procedure

Following Hunter and Schmidt (1990), we corrected observed correlations for sampling error. In addition to reporting the estimates of the mean true score correlations, we also reported variability in the correlations. Variability was indexed by both 95% credibility intervals and 95% confidence intervals around the estimated population correlations. Credibility intervals estimate the variability of individual correlations across studies, whereas confidence intervals estimate the variability around the estimated mean correlation.

We investigated two moderators: age and culture. We divided age into four levels to see whether the effects of humor styles on SWB are influenced by different developmental stages: childhood (6–12 years old), adolescence (12–18 years old), young adulthood (18–22 years old), and adulthood (older than 22). We observed cultures and ethnic regions including China, the United States, Canada, India, the United Kingdom, Australia, Hong Kong, Switzerland, Italy, Germany, Japan, and South Africa. The two research assistants and the first author coded the countries as being typically Western (−1), typically Eastern (1), or “other” (0) if they failed to categorize a culture into either Western or Eastern, such as Turkey and Israel. The interrater reliability was 1 for culture.

## Results

**Figure 2:**
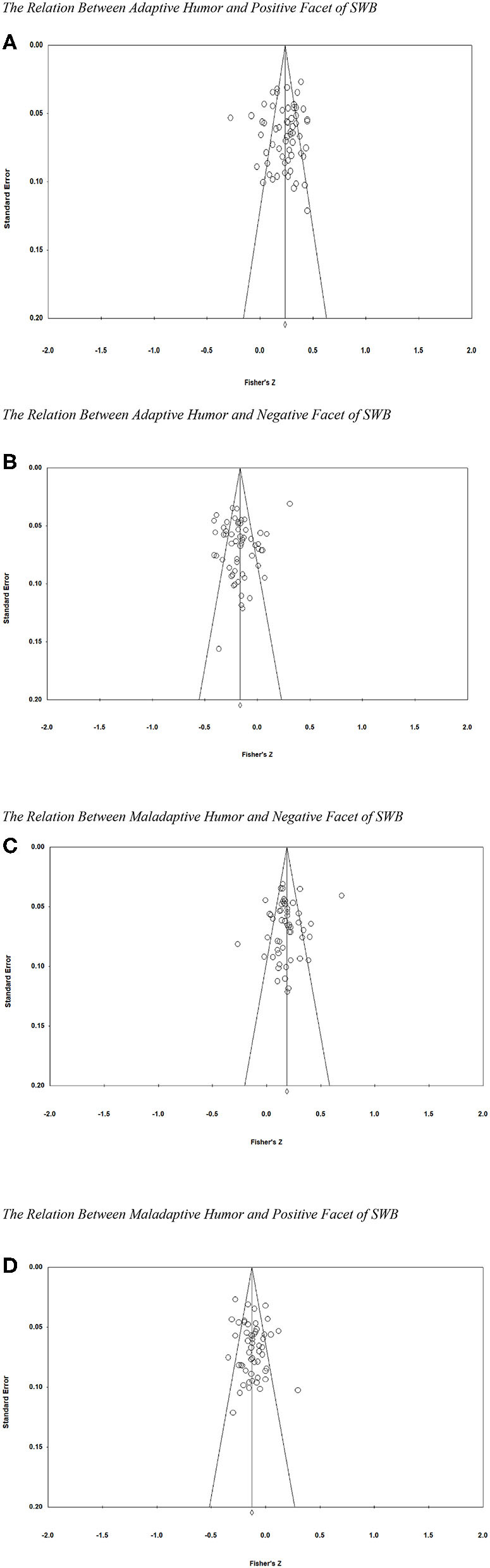
Funnel plot for publication bias test. **(A)** The relation between adaptive humor and positive facet of SWB. **(B)** The relation between adaptive humor and negative facet of SWB. **(C)** The relation between maladaptive humor and negative facet of SWB. **(D)** The relation between maladaptive humor and positive facet of SWB.

Table 1 shows the results of the meta-analyses relating humor styles to SWB. Adaptive humor was positively correlated with the positive facet of SWB (ρ = 0.227) and negatively associated with the negative facet of SWB (ρ = −0.124). The Kendall's tau tests results were τ(*N* = 63) = −0.04, *p* = 0.64 and τ(*N* = 59) = 0.14, *p* = 0.13, respectively, indicating non-significant publication bias. The funnel plots of publication bias test are displayed in Figures 2A,B for relations between adaptive humor and the positive facet of SWB and adaptive humor and the negative facet of SWB, respectively. In contrast, maladaptive humor was positively correlated with the negative facet of SWB (ρ = 0.181) and negatively correlated with the positive facet of SWB (ρ = −0.160). The Kendall's tau tests were τ(*N* = 56) = 0.08, *p* = 0.37 and τ(*N* = 59) = 0.02, *p* = 0.82, respectively, indicating non-significant publication bias. The funnel plots of publication bias test are presented in Figures 2C,D for maladaptive humor–negative SWB and maladaptive humor–positive SWB, respectively. Confidence intervals of the four relationships excluded zero, ensuring confidence that the average correlations are distinguishable from zero. However, the 95% credibility interval included zero for all four relationships, indicating that humor styles have varying relationships with SWB across studies. Sampling error, sample correlation, and population correlation explained only a small percentage of the variability in the correlations across studies. Across the four associations, only 13.71% of the variability in the correlations was explained.

**Table 1:** Meta-analysis of the relationship of humor styles to SWB.

**SWB (positive facet)**								
	**Average**				**95% CV**		**95% CI**	
	***k***	***N***	***r***	**ρ**	**Lower**	**Upper**	**Lower**	**Upper**
Adaptive humor	63	23,197	0.225	0.227^***^	−0.014	0.468	0.193	0.262
Maladaptive humor	59	21,361	−0.114	−0.124^***^	−0.284	0.036	−0.144	−0.089
**SWB (negative facet)**								
Adaptive humor	59	19,225	−0.167	−0.160^***^	−0.454	0.133	−0.212	−0.126
Maladaptive humor	56	18,602	0.173	0.181^***^	−0.032	0.394	0.141	0.214

*k, number of correlations; N, combined sample size; ρ, estimated true score correlation; CV, credibility interval; CI, confidence interval*.

*** *p < 0.001*.

In addition to studying the relationship between humor styles and SWB, we examined whether the relationship would vary among people of different ages and different cultures. However, age and culture failed to moderate the relationship between humor styles and SWB. Table 2 provides detailed moderation information.

**Table 2:** Moderation effects of age and culture.

	**Adaptive humor–SWB** **(positive facet)**	**Adaptive humor–SWB** **(negative facet)**	**Maladaptive humor–SWB** **(positive facet)**	**Maladaptive humor–SWB** **(negative facet)**
Moderators	χ^2^ (*p*)	χ^2^ (*p*)	χ^2^ (*p*)	χ^2^ (*p*)
Age	3.967 (0.411)	1.180 (0.947)	1.806 (0.771)	0.316 (0.997)
Culture	0.329 (0.848)	4.188 (0.123)	2.482 (0.289)	0.971 (0.615)

## Discussion

The current research aimed to investigate the humor and SWB relationship, as well as the moderating role of culture and age. Along with previous literature (e.g., Kuiper and Martin, 1993, 1998; Kuiper and Olinger, 1998; Lefcourt, 2001; Chen and Martin, 2007; Martin and Ford, 2018), we found that adaptive humor styles benefit whereas maladaptive humor styles undermine SWB. More important, culture did not moderate the relationship between humor styles and psychological well-being. No matter where they are from, people still benefit from adaptive humor and experience maladaptive humor as detrimental. In addition, we found that age did not moderate the humor and SWB relationship either. The assumption that adaptive humor is beneficial and maladaptive humor is detrimental to SWB holds up in the samples of children, adolescents, young adults, and adults.

### Relations Between Humor and SWB

Previous research on humor styles and SWB has loosely been conducted, randomly focusing on some aspects of SWB. Consistent with Diener and Lucas (1999) suggestions, SWB incorporates both cognitive judgments of one's life satisfaction and affective evaluations of mood and emotions. In the current research, we included a wider scope of SWB measures, such as anxiety, distress, subjective happiness, stress, positive affect, negative affect, depression, optimism, self-esteem, life satisfaction, school satisfaction, job satisfaction, loneliness, extraversion, neuroticism, flourishing, and so forth. The results provide a systematic view of the humor and SWB relationship, confirming that adaptive humor benefits whereas maladaptive humor undermines SWB.

### Culture Effect on the Relation Between Humor and SWB

The results on cultural difference in the relationship between humor styles and SWB are contradictory. For example, Chen and Martin (2007) found that in both the Chinese and Canadian samples, adaptive humor styles were negatively correlated, whereas maladaptive humor styles were positively correlated with the scores of SCL-90. Schneider et al. (2018) meta-analysis found that an aggressive humor style was negatively associated with self-esteem and was positively associated with depression for Easterners, but not for Westerners. Consistent with research on Western samples, we found that adaptive humor styles benefit whereas maladaptive humor styles undermine SWB (Kuiper and Martin, 1993, 1998; Kuiper and Olinger, 1998; Lefcourt, 2001). However, we did not find the moderating effect of culture on the relationships between humor styles and SWB, though previous research suggests that Easterners' humor perception and usage is different from Westerners' (e.g., Rudowicz and Yue, 2002; Yue, 2010, 2011; Yue et al., 2016a). The absence of culture effect may be due to three reasons: First, compared with Westerners, Easterners have complicated beliefs about humor, which does not simply contradict the beliefs that Westerners hold. For example, Chinese society tends to follow Confucian philosophy, which deems humor degrading and frivolous (Rudowicz and Yue, 2002; Yue, 2011). In the meantime, Chinese culture also follows Taoist and Buddhist teachings, which highlight the humorous spirit as a witty and harmonious interaction with nature (Yue, 2010, 2011). The conflicting beliefs might have made Chinese people ambivalent toward humor, disdaining and appreciating humor simultaneously (Yue, 2011). Moreover, culture is not a static construct and is subject to the influences of many factors (Greenfield, 1997; Oishi and Graham, 2010; Cai et al., 2019). For example, Inglehart and Oyserman (2004) found that economic growth drives a shift from collectivism to individualism. The dynamic changes of culture make it possible that the results on cultural difference in the relationship between humor styles and SWB are not consistent. How culture as a dynamic process influences the relationship between humor styles and SWB merits future investigations.

Yue (2011) summarized three ambivalent attitudes toward humor among Chinese: First, Chinese value humor but devalue self-humor. He argued that Confucian puritanism and conservatism regards humor as inferior forms of expression. Therefore, to protect their social status, Chinese people tend to be reluctant to express humor. Second, being humorous is not associated with being an orthodox Chinese person. This is also consistent with the doctrine of Confucianism, which assumes humor represents intellectual shallowness and social informality. Thus, the Chinese are less likely to regard humor as an ideal personality trait (Yue et al., 2016a). Third, the importance of humor varies according to the individual. Given that humor is not an ideal Chinese personality trait, Chinese people tend to believe humor should be left to specialists (e.g., entertainers, comedians) rather than just anyone (Yue et al., 2016a). Similarly, Jiang et al. (2019) argued that due to dialectic thinking style, Chinese people tend to show contradictory attitudes toward humor that are simultaneously positive and negative. Taken together, such ambivalent belief may lead to mixed findings on a humor effect among Chinese (Sun et al., 2009; Jiang et al., 2011; Yue et al., 2017), which in turn weakens the moderating role of culture on the relation between humor and SWB.

Second, another reason why we failed to observe a culture effect on the relation between humor style and SWB might be that the number of studies conducted in Eastern culture is relatively small. In this meta-analysis, Eastern culture studies took only a percentage of 23.7–28.8% in all four types of humor and SWB links. Thus, future investigations providing more empirical findings from Eastern culture will be helpful for identifying whether the moderating role of culture exists in the humor and SWB relationship.

### Age Effect on the Relation Between Humor and SWB

Past research has provided rather mixed findings regarding how age affects the relation between humor and SWB. Some argue for age differences (e.g., Bell et al., 1986; Erickson and Feldstein, 2007), whereas others disagree (Führ, 2002), with no consensus (e.g., Feldman et al., 1996; Erickson and Feldstein, 2007). Our meta-analysis seems to be supportive of the contextual hypothesis (Folkman and Lazarus, 1980; McCrae, 1984) by indicating that individuals at all life stages benefit from adaptive humor and suffer from maladaptive humor. However, we should interpret this finding cautiously. The results that age did not moderate the relation between humor and SWB may be attributed to the way we coded the data. In current research, given parts of the studies did not report age clearly, to obtain a consistent data mode, we coded participants by age into four groups: children (6–12 years old), adolescents (12–18 years old), young adults (18–22 years old), and adults (older than 22). In doing so, we simplified age data as a categorical variable, which might weaken its potential effects on the relation between humor and SWB. In addition, our meta-analysis has unbalanced age distribution (i.e., 3.9% children, 1.6% adolescents, 60.9% young adults, and 33.5% adults), which may also be responsible for the null effect of age. Future research should recruit participants from a wide range of ages or employ longitudinal research design to test how age influences the relation between humor and SWB.

## Limitations and Future Directions

Our meta-analysis showed that humor that is affiliative and self-enhancing is positively associated with SWB, whereas humor that is aggressive and self-defeating is negatively associated with SWB. Culture and age do not moderate the relationship. However, future investigation should be aware that our research has some limitations.

First, we could draw no causality conclusions. Most research on humor and SWB is cross-sectional. Our meta-analysis was also unable to provide causal evidence. Humor has implications on SWB, and SWB in turn could affect how one uses humor. To clarify how humor affects SWB, future research should employ well-designed experimental methods to test the humor and SWB relationship.

Second, we coded Western, Eastern, and “other” cultures according to regions and nationalities, which captured cultures by demographic regions (e.g., Chen and Martin, 2007; Yue et al., 2010; Hiranandani and Yue, 2014). However, this index is somewhat inaccurate, especially in the trend toward globalization and cultural mixing (Cai et al., 2019). Future research should investigate cultural dimensions empirically to better understand how culture affects the relationship between humor and SWB. Moreover, due to the limits of our research team, we focused only on studies published in English and Chinese. Such linguistic scope may weaken our interpretation of cultural differences. Future research should include additional publications in other languages and unpublished dissertations from the under-represented demographic region.

Third, we find that culture and age are not moderators: humor and SWB have similar qualitative relationships for Westerners and Easterners, the young and the old. However, we could not rule out possible quantitative differences. For example, mainland Chinese tend to use more affiliative and less aggressive humor than bicultural Hong Kong students do. Similarly, adaptive humor is more strongly and positively associated with optimism for mainland Chinese students (e.g., Yue et al., 2010, 2014b). In addition, although for people of all ages humor that is affiliative and self-enhancing is beneficial and humor that is aggressive and self-defeating is detrimental, specific humor styles might emerge, and mature during particular developmental stages (Erickson and Feldstein, 2007). Therefore, it is worth investigating how the relationship between humor and SWB differs quantitatively for people with different cultural backgrounds and ages.

## Conclusion

Humor is ubiquitous (Fry, 1994), but people from various cultural backgrounds may perceive and use humor differently. Nevertheless, humor has consistent relationships with SWB across cultures and ages; that is, humor that is affiliative, and self-enhancing will enhance SWB. In contrast, humor that is aggressive and self-defeating will damage SWB.

## Data Availability Statement

The dataset is available on request. Requests to access these datasets should be directed to Feng Jiang, fengjiang0205@gmail.com.

## Author Contributions

FJ, SL, and TJ conceptualized the idea. HJ helped to collect the data. FJ and SL analyzed the data. TJ drafted the manuscript. FJ, SL, and HJ reviewed and revised the manuscript. All authors contributed to the article and approved the submitted version.

## Conflict of Interest

The authors declare that the research was conducted in the absence of any commercial or financial relationships that could be construed as a potential conflict of interest.
